# Reflections on adversarial collaboration from the adversaries: was it worth it**?**

**DOI:** 10.1007/s11186-025-09634-2

**Published:** 2025-06-16

**Authors:** Calvin Isch, Philip E. Tetlock, Cory J. Clark

**Affiliations:** 1 University of Pennsylvania, 3620 Walnut St, Philadelphia, PA 19104 USA; 2 New College of Florida, 5800 Bay Shore Rd, Sarasota, FL 34243 USA

**Keywords:** Adversarial collaboration, Metascience, Scientific method, Research methods, Collaboration

## Abstract

There is much enthusiasm, in principle, for adversarial collaborations (ACs), a scientific conflict resolution technique that encourages investigators with clashing models to collaborate in designing studies that test competing predictions. Adversarial collaborations offer the promise of breaking deadlocked debates, resolving disputes, and providing a deeper, more comprehensive understanding of a research domain. In practice, however, adversarial collaborations are more the exception than the rule, and there is almost no evidence on how scholars who have ventured into ACs assess the experience. To understand these perspectives, we surveyed and interviewed 29 scholars who participated in 13 AC projects. The data revealed that interpersonal conflicts were generally minor, that these projects required more upfront effort than typical collaborations, but benefited from high-quality results and more thoughtful post-publication debates. Rather than producing a clear “winner,” the most common outcome was a deeper understanding of the problem space through the integration of opposing perspectives. Although the generalizability of these findings is limited by a sample consisting only of scholars who completed an AC, they nonetheless highlight the value of ACs as a tool for advancing scientific inquiry and offer practical guidance for scholars and journals exploring this approach.

*“Science and scientific objectivity do not (and cannot) result from the attempts of an individual scientist to be ‘objective’, but from the friendly-hostile co-operation of many scientists.”* —Karl Popper, The Open Society and Its Enemies (2012, p. 489).

## Introduction

The past decade has brought transformative introspection to the social sciences. The replication crisis has revealed that many findings fail to hold under even the most shallow scrutiny—direct replication (Open Science Collaboration, 2015). Analytical flexibility allows opposing results to be derived from the same data (Ioannidis, 2005; Silberzahn et al., 2018), and the disconnect between verbal interpretations and statistical outcomes has become a growing concern (Yarkoni, 2022). These issues partially derive from the tension presented in the opening quote: science thrives on adversarial critique yet often prioritizes individuals serving as “objective” promoters of their theories and rarely, if ever, requires them to resolve persistent contradictory claims. Consequently, proponents of low-quality theories must die for science to progress (Planck, 1949).

Adversarial collaborations (ACs) have been heralded as a potential remedy, promising rigorous construct operationalizations, sound analyses, and measured interpretations. Indeed, as far back as 2003, Nobel Laureate, Daniel Kahneman asserted that ACs “would contribute to an enterprise that more closely approximates the ideal of science as a cumulative social product” (Kahneman, 2003, p. 730). Yet, their promise remains almost entirely theoretical (Ceci et al., 2024). Can ACs resolve conflicts and improve research quality? And exactly how annoying are they to participate in? To explore these questions, we surveyed scholars who had engaged in ACs. Their reflections reveal a nuanced picture: while challenging to initiate and complete, ACs often save time during peer review, result in higher-quality research (as perceived by collaborators), and leave participants enthusiastic for future collaborations.

## Scholarly business as usual

In the course of normal science, scholars often identify theories and results that seem to conflict with one another. For example, a scholar finds that people systematically overestimate their abilities (Dunning, 2011), while others argue that people underestimate them (Chrousos & Mentis, 2020). Some find that people place disproportionate weight on initial values, anchoring to arbitrary numbers (Furnham & Boo, 2011), whereas others show that people frequently neglect base rates even when they are explicitly provided (Pennycook et al., 2022). A group may find a small to medium effect size for nudging behavioral interventions (Mertens et al., 2022), while others can make different assumptions with the same data and find no effect whatsoever (Maier et al., 2022). While disagreement and debate are essential parts of the scientific process, existing norms often result in incommensurate theories and a set of findings which, while each interesting and defensible on their own, cannot be “put together” or made sense of in aggregate (Newell, 1973; Meehl, 1990; Almaatouq et al., 2024). Consequently, scholars continue generating findings and results in separate worlds, failing to resolve disagreement.

Part of these conflicting findings may result from poor methodology. Over the past decade, the replication crisis has highlighted systemic issues in the social sciences, revealing that a substantial portion of psychological studies fail to replicate under rigorous testing (Open Science Collaboration, 2015). Adding to this, “Many Analysts” studies demonstrate that researchers analyzing the same dataset often arrive at divergent conclusions. Differences in analytical techniques can dramatically change estimates of effects (Silberzahn et al., 2018) or result in predictions that vary widely (Salganik et al., 2020). These issues are further exacerbated by researchers’ decisions in experimental design and construct operationalization, which can lead to conflicting results, including effect sizes pointing in opposite directions (Huber et al., 2023). Some conflicts are mere illusions because different scholars use the same words to describe different constructs or different operationalizations to describe the same construct, yet nonetheless can stalemate debates because participating scholars never bother to clarify the nature of their disagreement. Together, these realities emphasize the need for a more robust approach to addressing empirical challenges.

Open science norms—such as preregistration, data sharing, and collaborative research practices—have made significant strides in improving transparency and reproducibility (Nosek et al., 2022), yet they do not ensure appropriate considerations of the boundaries under which a phenomenon is likely to occur. Similarly, integrative experiments, crafted to explore the relevant parameter space for a given phenomenon, may offer valuable insights into boundary conditions (Almaatouq et al., 2024), but when these teams lack individuals with competing interests and perspectives in the design process, they may produce operationalizations that poorly capture important parameters (Clark et al., 2024). Further, neither of these approaches adequately addresses the generalizability crisis, a problem brought about by scholars relying on narrowly defined operationalizations that fail to extend beyond their immediate context while still making broad verbal claims in their papers (Yarkoni, 2022). In summary, these reforms, which rely on teams acting as “objective” individuals, cannot address deeper conceptual problems.

ACs offer a promising solution by embedding diverse perspectives throughout the research process, from research design to analysis to interpretation. By fostering fair construct operationalization, rigorous methodology, and circumspect verbal descriptions of findings, ACs can address both methodological weaknesses and conceptual shortcomings, improving the quality and validity of social science research (Clark et al., 2022). While adversarial collaborations have long been promoted by luminaries in the field (Kahneman, 2011; Mellers et al., 2001), no empirical work has systematically assessed how AC participants viewed their experiences. Here we conducted a survey of scholars who had attempted this technique to answer five questions. (1) How do people get involved in ACs? (2) Are ACs much harder than typical projects? (3) Do scholars change their minds throughout the process? (4) Are ACs worth the effort, producing higher quality papers? (5) What advice can participants provide for future endeavors?

### Method

*Sample*. In spring 2022, we conducted a systematic literature search to identify all published ACs. Our search was performed on Google Scholar using the keyword “Adversarial Collaboration.” Papers were included if they reported novel empirical findings produced by competing scholars, and the search was terminated after encountering 50 consecutive articles that did not meet the inclusion criterion. We supplemented this set with additional papers recommended to us that involved adversarial collaborations without explicitly using the term, as well as two near-completion adversarial collaborations that had not yet undergone peer review. Finally, we identified one AC that was ultimately unsuccessful, as the authors abandoned the effort to produce a joint publication and instead published separate papers. This last paper is not listed in Table 1 to preserve anonymity. Note that our list is likely missing adversarial collaborations that never made it to publication, that did not call themselves an AC, and that were published after our search.

**Table 1:** Adversarial collaboration publications whose authors were contacted to participate in our study

Authors	Title
Latham et al., 1988	Resolving scientific disputes by the joint design of crucial experiments by the antagonists: Application to the Erez–Latham dispute regarding participation in goal setting
Gilovich et al., 1998	Varieties of regret: A debate and partial resolution
Mellers et al., 2001	Do frequency representations eliminate conjunction effects? An exercise in adversarial collaboration
Bateman et al., 2005	Testing competing models of loss aversion: An adversarial collaboration
Schlitz et al., 2006	Of two minds: Sceptic‐proponent collaboration within parapsychology
Cadsby et al., 2008	Step return versus net reward in the voluntary provision of a threshold public good: An adversarial collaboration
Corrigan et al., 2012	Repeated rounds with price feedback in experimental auction valuation: An adversarial collaboration
Matzke et al., 2015	The effect of horizontal eye movements on free recall: A preregistered adversarial collaboration
Van Dessel et al., 2017	Mechanisms underlying approach-avoidance instruction effects on implicit evaluation: Results of a preregistered adversarial collaboration
Kerr et al., 2018	Addressing replicability concerns via adversarial collaboration: Discovering hidden moderators of the minimal intergroup discrimination effect
Alempaki et al., 2019	Reexamining how utility and weighting functions get their shapes: A quasi-adversarial collaboration providing a new interpretation
Cowan et al., 2020	How do scientific views change? Notes from an extended adversarial collaboration
Koch et al., 2020	Groups' warmth is a personal matter: Understanding consensus on stereotype dimensions reconciles adversarial models of social evaluation
Melloni et al., 2021	Making the hard problem of consciousness easier
Stern & Crawford, 2021	Ideological conflict and prejudice: An adversarial collaboration examining correlates and ideological (a)symmetries
Bowes et al., 2023	An Adversarial Collaboration on the Rigidity-of-the-Right, Rigidity-of-Extremes, or Symmetry: The Answer Depends on the Question
Martel et al., 2024	On the efficacy of accuracy prompts across partisan lines: an adversarial collaboration

We reached out to all authors and participants in these projects (k = 18; N = 87), sending a request to either complete our survey or arrange a time for an interview. A total of 29 scholars, representing 13 projects, participated in our study, the majority of whom (N = 24) filled out the survey. Notably, all participants came from projects that resulted in publication or were nearing completion; no scholars who began but did not complete an AC responded to our inquiries. This sample limits the generalizability of our findings, as those who followed through with an AC may differ in important ways, such as disposition or motivation, from the broader academic population. Nonetheless, we believe their experiences offer valuable insights for others considering this approach. Scholars were informed prior to participation that their information would be kept confidential to encourage open and honest reflections. Consequently, we do not disclose the identities of the participants or attribute specific quotes and responses to projects. Most participants were adversaries in these projects, though three had served as arbiters (also called moderators).

*Survey and Interview:* Our survey, containing 10 items, aimed to explore several key themes (See Table 2). For those who engaged in interviews, we crafted semi-structured items that addressed the same questions while allowing for greater flexibility to explore specific ideas from each project.

**Table 2:** Survey questions presented to all participants in our study and associated themes

Theme	Question
Getting involved	What made you decide to do an adversarial collaboration?
	How did you know your collaborator before beginning the adversarial collaboration? How different were your approaches at the beginning of the project?
Challenges, conflict, and opportunities	Compared with your other projects of a similar scope, how was the adversarial collaboration experience different? Was it more challenging or easier than more typical projects?
	Were there any conflicts during the collaboration? If so, what were those? Why do you think they arose? How did you resolve them?
Belief updating	Did either of you shift your priors at the conclusion of the adversarial collaboration? Why or why not?
	From your perspective, was your adversary more interested in defending their hypothesis or better understanding the truth? Please explain and also reflect on your own interests
Quality of the results	How would you rate the scholarly quality (regarding e.g., the likelihood that your findings will replicate/generalize, the novelty of your approach, the importance of the projects contribution) of your adversarial collaboration compared with your other projects of a similar scope?
	Would you do an adversarial collaboration again? Why or why not?
Advice for success	What does a successful collaboration look like? What specific steps did you and your collaborator take to make this collaboration successful?
	What advice would you give individuals considering an adversarial collaboration?

*Analysis:* We analyzed these responses qualitatively, following an approach rooted in grounded theory. Responses were compiled and classified iteratively to construct our final coding scheme for each question. We then developed overarching themes that became apparent across items and participants. While our initial questions were guided by specific expectations, we remained open to new insights and ideas that emerged from the scholars’ responses, particularly given the diverse levels of involvement many had in the projects.

## Results

Our findings are structured around five key insights that address the questions posed in the introduction. We provide both quantitative and qualitative examples for each section. Quotes from participants are provided with only minor grammatical corrections (See Table 3).

**Table 3:** Summary table with key insights and illustrative quotes

Insight	Quotes
Serendipity guides the formation of ACs so far, but more systematic approaches may be possible	“We had two different teams of researchers who had published work getting conflicting results.”
	“A collaborator suggested this as an alternative to him writing a comment on our paper.”
	Our project emerged out of “a bet with a close colleague”
	“I was really attracted by the idea of several teams confronting their points of view.”
	*Author Insight*: Journals or conference venues could organize ACs for greater systematicity
ACs are more challenging than typical collaborative projects, though the effort is often seen as more effective and rewarding and may also hasten peer review and improve post-publication debate	“The methodological choices are discussed at length, and it is very complex to reach agreement.”
	“It was more anxiety-provoking at the outset because the coauthor who initiated the AC was roughly 10 times more accomplished than me.”
	“This need to justify one's choices is one of the advantages…because it makes it possible to become aware of choices that may be seen as arbitrary by another team.”
	“Logistically [ACs are] more challenging but ultimately more rewarding scientifically and methodologically.”
	“Due to the choice of the mediator, the experience was highly collegial—easier.”
Scholars rarely abandon their original theory but often arrive at a more nuanced understanding of the problem space	“Perhaps both sides moved a little more towards a middle ground.”
	“Our studies allowed us to know when my collaborator’s intuition holds and when mine (and my prior findings) hold”
	“We should have measured this (but didn't). We shifted our opinion, and the adversaries as well.”
	“Yes, [we] definitely [changed our minds]. In our part of the AC we found out that a certain analytical decision we had taken was partially responsible for the drastic contradiction to the existing model.”
	“Yes, although we left in the paper areas of open disagreement.”
	“We all started from a position of mild confusion/curiosity and ended up at one with more clarity.”
	The data supported our model, so we “didn't have to change our views. The other team probably did, but they were slow to come around.”
	“No, people don’t seem to change their minds much. They just open them a little wider.”
	“The results were not statistically significant in either direction, so I don’t think anyone changed their mind.”
Conflicts are surprisingly rare	“Conflicts have been very small…[and they] are usually resolvable over email discussion and back and forths on google docs.”
	“No conflicts to speak of. There are many ways to approach empirical analysis, for example, but we left that to one of our neutral coauthors, minimizing the potential for conflict.”
	There were “many disagreements, mainly about interpretations of data patterns… They were resolved with discussions and precise analyses of the available data.”
	“Sometimes surprising results were met with mild defensiveness, but this was overcome with working through the conclusions with copious amounts of coffee and cake.”
Most scholars believe their final product was of very high quality and would try ACs again	“Simple answer: Better. More effort, more thought, advancement by competition leads to better science”
	The quality was “higher than average, though it will probably be ignored by those who prefer nice easy experiments which support prior positions.”
	“I apply to all my research the same highest scientific standards. In the case of this AC, the contributions build on a number of replications and, thus, de facto it stands on a larger set of empirical evidence, which is bound to make our inference more robust.”

### Serendipity guides the formation of ACs so far, but more systematic approaches may be possible

Scholars were drawn to ACs to address and resolve conflicting findings that arose during the course of scientific research. Many described their motivation as stemming from significant theoretical or empirical disagreements, with one noting, “We had two different teams of researchers who had published work getting conflicting results.” Others referred to their involvement as a response to “severe disagreement,” “scientific conflict,” or “completely opposite predictions.” These collaborations offered a structured way to tackle such challenges collaboratively.

While disagreement was a common motivator for engaging in ACs, many scholars cited other reasons. Some emphasized pragmatic considerations: “We thought it would increase the likelihood of acceptance and impact of the article,” or improving the productivity of criticism: “A collaborator suggested this as an alternative to him writing a comment on our paper.” Others expressed curiosity, describing the process as “a bet with a close colleague” or “excitement about solving a research problem/discovering the truth.” One early career scholar noted, “I was really attracted by the idea of several teams confronting their points of view,” seeing it as an opportunity for theoretical and methodological enrichment. Still, some offered less idealized rationales, such as following the lead of a principal investigator or recognizing, “…other people were doing a lot of the leg work, and so it wouldn’t take a ton of my time.”

**Fig. 1:**
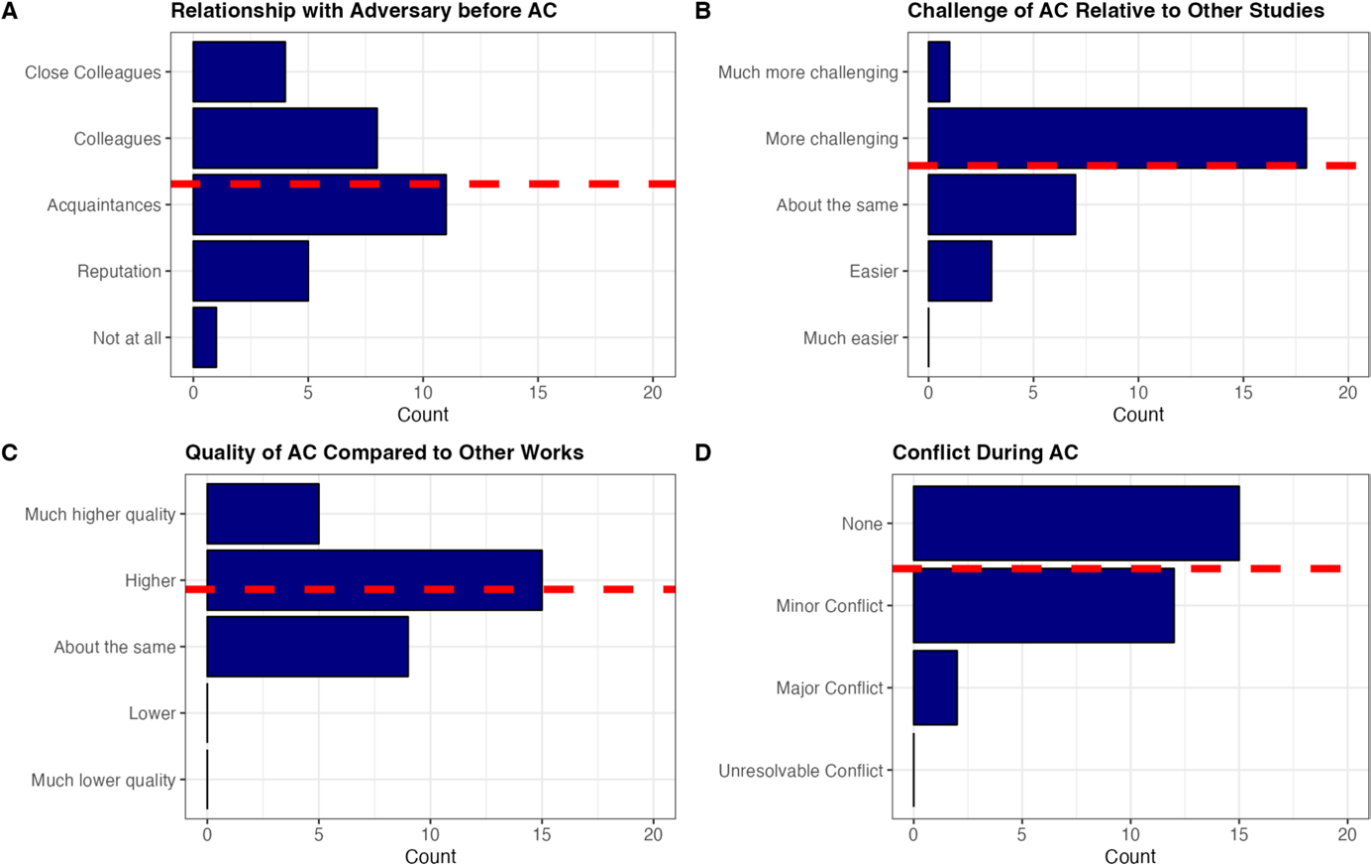
Bar plots displaying our coding of four questions. The dashed red line represents the mean, calculated by treating each response category as numeric. (Panel **A**) There is a large degree of heterogeneity in the level of familiarity between participants and their adversaries before the AC. (Panel **B**) ACs tend to be more challenging than other projects of similar scope. (Panel **C**) ACs often produce higher quality work than traditional projects. (Panel **D**) AC conflicts tend to be rare and minor

Finally, we asked participants about their familiarity with their adversaries before engaging in the project. Responses varied widely, ranging from being entirely unfamiliar with their adversaries’ work, to knowing them only by reputation, to being close colleagues (See Fig. 1a). In short, there was no prototypical relationship between collaborators before beginning the AC. This serendipitous nature prompts consideration of whether a more systematic approach could improve their effectiveness. For instance, if journals or organizations implemented standardized methods for identifying conflicts and assembling teams, it could expand the scope of ACs and facilitate the resolution of more scientific disputes. We explore this idea further in the discussion.

### ACs are more challenging than typical collaborative projects, though the effort is often seen as more effective and rewarding, and may also hasten peer review and improve post-publication debate

One common concern about ACs is that they may be significantly more challenging than typical research projects. Scholars worry that involving adversaries could make it nearly impossible to reach consensus on measurement strategies or analytical approaches. When asked about the level of difficulty of ACs, most participants agreed that these collaborations were more challenging (see Fig. 1b) and required more time, often due to differences in operational definitions and lengthy discussions around methodological choices: “We first discovered some differences in the operationalization of our research variables,” one scholar remarked, while another explained, “The methodological choices are discussed at length, and it is very complex to reach agreement.”

Despite these challenges, scholars frequently highlighted benefits, noting increased impact and recognition of their work: “However, it is much more impactful,” and “I felt I got more appreciation.” Others emphasized how the rigorous justification of research decisions strengthened the project, with one participant stating, “This need to justify one’s choices is one of the advantages…because it makes it possible to become aware of choices that may be seen as arbitrary by another team.” Another observed, “We had to all agree up front what a good project looked like, as opposed to all arguing after the fact about data that one or the other (but not both) sides had collected.” Finally, one scholar compared the AC approach to a more common method from the literature: “I found the article-comment-reply experience much more confrontational, much less constructive, and ultimately much less satisfying than adversarial collaboration.” These reflections underscore a key insight: while ACs demand substantial upfront investment, this effort can save time and reduce conflict in later stages, ensuring greater clarity and mutual understanding.

### Scholars rarely abandon their original theory but often arrive at a more nuanced understanding of the problem space

When asked whether their adversarial collaboration led them or their adversary to shift beliefs, participants rarely provided straightforward answers. Instead, their responses were often nuanced, suggesting that “perhaps both sides moved a little more towards a middle ground” or that “all collaborators believed we moved the field forward.” To better understand these responses, we categorized them into three broad groups and found that ~ 34% reported belief updating, 24% reported no belief updating, and ~ 41% reported that it was complicated.

Among those who described belief updating as ‘complicated,’ some challenged the premise of the question itself. One remarked, “I think this is the wrong question to ask. Science is about theory and evidence. Scientists’ priors are not relevant for science!” Others reframed the discussion, emphasizing that the collaboration did not decisively prove one theory correct over another. Instead, they highlighted more incremental progress, noting that “our studies allowed us to know when my collaborator’s intuition holds and when mine (and my prior findings) hold” or reflecting that “Each theory has evolved a bit.” These responses suggest that while ACs may not always resolve debates, they can refine theories and advance understanding.

When beliefs did not shift, scholars cited strong evidence for their original hypothesis or inconclusive results: “The results were not statistically significant in either direction, so I don’t think anyone changed their mind.” Of course, it is not clear whether such outcomes warrant little or no belief updating, and future work should further explore whether scientists more broadly tend not to update beliefs in response to findings that do not support or indeed oppose their own hypothesis. If so, this may suggest broader challenges for the scientific community.

Aligned with this concern, scholars often claimed that the results of ACs supported their own perspective more than their adversary’s. When reporting that opinions changed, they either asserted that both parties shifted their views to a similar extent or that their adversary moved closer to their original position. Notably, five scholars expressed that their adversary’s change was insufficient given the new evidence. In contrast, no scholar reported that the results aligned more strongly with their opponent’s perspective. This pattern may partly reflect participation bias if scholars whose ACs supported their theories were more likely to participate in our study. However, to date, we are not aware of any AC where one side openly admitted thorough defeat. ACs might tend not to produce obvious victories. Or, these patterns may reflect motivated reasoning can persist even within a highly controlled collaborative framework like ACs. A greater quantity of ACs will be necessary to identify clearer cases of victory and the conditions that facilitate such outcomes.

### Conflicts are surprisingly rare. Most scholars believe their final product was of very high quality and would try ACs again

ACs are, by design, adversarial, and so individuals often raise concerns about potential conflict along the way. But how often did adversarial collaborators observe interpersonal conflict? We categorized participants’ responses into four categories: (1) No conflict, (2) Minor conflict, (3) Major conflict, and (4) Unresolvable conflict. As displayed in Fig. 1d, the modal response was no conflict whatsoever. When scholars did report conflict, it tended to be minor and resolvable through the use of an arbiter. Thus, ACs may be less conflict-prone than many assume (although possibly more conflict-prone than a typical project). The majority of scholars also reported that their AC was indeed of higher quality than their other published work. Not a single scholar reported lower quality (see Fig. 1c).

Perhaps the ultimate test of whether an individual believes in ACs is whether they are willing to participate again. Almost all scholars said they would (N = 27). Many of these were eager (N = 18), but others were more hesitant (N = 9) and stated that it would take very particular circumstances. For example, “I would be very careful in selecting who I would want to work with.” Others mentioned the importance of addressing an appropriate question: “Yes, if a reputable researcher challenged my interpretation of an experiment I had run, and if there was an experimental design to discriminate between the hypotheses.” One person said they would not do it again, and another did not answer this question because they had retired.

### For success: choose your adversaries wisely, involve a neutral arbiter and early career scholars, and keep extensive documentation

When asked about steps to ensure a successful AC and advice for such projects, participants’ responses often aligned with recommendations from existing publications (see Clark et al., 2022; Cowan et al., 2020). One of the most common pieces of advice was to *carefully choose adversaries*. Scholars emphasized that not everyone is suited for these projects, highlighting the importance of intellectual humility. Ideal collaborators were described as having “a respectful approach to the others’ ideas,” a “willingness to learn from adversaries,” and being “genuinely open and curious.”

A second key recommendation was the involvement of a neutral third-party arbiter. This individual should be “trusted and respected by both parties” to “help settle design disputes” and maintain fairness throughout the process. Third, participants stressed the importance of thorough documentation. This includes pre-project agreements that serve “almost as a contract” to hold collaborators accountable, as well as detailed, transparent documentation during the project in line with open science practices. For example, scholars emphasized the need for “clearly articulated methods and agreed-upon protocols” and noted that “the methodology must be well pre-registered beforehand.” Finally, many participants highlighted the value of involving individuals at different career stages. Senior scholars bring vested interests and ensure fair representation of ideas, while junior researchers, who are often less committed to existing theories, can help resolve conflicts and are well-positioned to analyze the data. By integrating these elements, future ACs can increase their likelihood of success (See Table 4 for recommendations with supporting quotes).

**Table 4:** Recommendations for successful ACs with illustrative quotes

Recommendation	Quote
Choose your adversary wisely	[Effective] “adversaries have to have a common commitment to the truth and a willingness to not just engage genuinely with the collaboration, but to learn from their adversaries.”
	“Ingredients [for success]: 1) Respect for the other group’s work 2) Intellectual Humility 3) Shared understanding of science.”
	“The most successful ACs involve scholars who are open, calm, friendly, and generous to their collaborator.”
Be a good adversary yourself	“Rather than focusing on the opponent and fearing how they might behave in an AC, focus on your own behavior and contributing to an interpersonal culture of respect and openness.”
	“Check your ego at the door and remember that you're a scientist.”
	“Hold your beliefs lightly. Stay open to unexpected outcomes. Practice social intelligence. Be flexible. Have humor. Show respect.”
Involve a neutral arbiter and early career scholars	Involve “a third independent party who can write an independent viewpoint.”
	It is important to have an arbiter “who both sides can trust and who is capable of keeping calm in crisis and who can effectively negotiate and find mutually acceptable compromises.”
	We agreed that “a junior colleague of mine would spend some time in their lab and collaborate with one of their junior colleagues to come up with studies that might explain why our results differ so systematically.”
Keep extensive documentation including pre-project agreements and pre-registrations	“Be meticulous about notes, predictions, beliefs, conditional beliefs and write everything down!”
	“We all agreed going in that we would proceed with the paper regardless of who's model was supported.”
	“Precise pre-registrations [and] data openness” are critical for success
Designate individuals to ensure steady progress	“The determination and effort of the most junior co-authors helped immensely.”
	“Having incentives for multiple people on both sides at various career stages” is essential for success
	"The arbiter needs to motivate collaborators when things get stalled."
Journals or conferences could organize ACs for greater systematicity	*Author Insight*: If journals or organizations adopted standard processes for identifying conflicts and forming teams, ACs could be more widely used to address scientific disagreements

### Additional insights from Arbiters

Our three arbiter respondents provided additional insights into the unique challenges and opportunities of their role in ACs. First, arbiters emphasized their critical role in mitigating conflict. They noted that maintaining a level head during heated moments is essential, including occasionally pausing meetings to diffuse tensions. In some instances, arbiters reported the need to split collaborators into separate email threads to facilitate negotiations independently. Arbiters also mentioned the need to develop creative solutions throughout the process; for example, one arbiter blinded herself to the conditions before conducting the analyses and writing the results to avoid potential partiality. Proactive conflict management from arbiters may explain why AC participants rarely reported overt conflict.

Second, arbiters highlighted their responsibility in maintaining momentum. ACs involve coordinating “lots of people with busy schedules,” making it vital for the arbiter to consistently push collaborators to stay engaged and meet deadlines.

Third, arbiters mentioned that another key to success, aligned with choosing your adversary wisely, was to be a good adversary yourself. “Rather than focusing on the opponent and fearing how they might behave in an AC, focus on your own behavior and contribute to an interpersonal culture of respect and openness… If you treat your adversaries well, they will return the favor.” However, like adversarial participants, they also noted that personality matters a lot and that not all scholars seem capable of ACs.

Aligned with the insights from collaborators, arbiters observed that ACs often produced higher-quality work with nuanced results. One arbiter attributed this to the frequent pretesting of materials and the use of a “multiverse approach,” which enabled multiple analyses across different contexts. As a result, findings generally failed to reflect “overwhelming victory for one side” and instead highlighted that both sides were “a bit right and a bit wrong in different contexts.” It is perhaps this reason that scholars didn’t change their minds a great deal or that these projects rarely seemed to provide a definitive winner. As one arbiter of several ACs observed, “I don’t think I have seen anybody entirely change their mind, but the results so far have not warranted that. The results tend to show both sides were perhaps exaggerating beforehand.”

Finally, arbiters appreciated the fully integrated outsider’s view of a research project, from which they could observe the decisions each team would have made if they had not been constrained by adversaries. From this unusual perspective, they could see how different teams could make very different choices that would lead to confident and contradictory results and how the presence of adversaries resulted in more carefully crafted methods and more restrained conclusions.

## Discussion

Through surveying and interviewing those who had completed ACs, we identified several lessons that shed light on the unique dynamics and potential for these projects to advance scientific inquiry. On one hand, ACs tended to produce higher quality outputs, conflicts were rare, and scholars frequently felt they advanced the understanding of a research field. On the other, ACs were harder than other projects, scholars never fully embraced their opponents’ perspectives, and some scholars might lack the temperament to complete ACs successfully. These findings highlight the promise of ACs and offer actionable insights for fostering their success, including careful selection of collaborators, involvement of neutral arbiters and early career scholars, and rigorous documentation throughout the process.

In addition to these lessons, it is important to address a common misconception about ACs. These projects are often construed as attempts to declare a definitive “winner” or “loser” in a scientific debate, but many theories in the social sciences are not universally true or false but instead hold validity under specific circumstances. As one scholar observed, “I feel as though it really pushes the idea that there are two (and only two) ‘sides’ to an argument when I don’t view things that way.” In complex problem spaces, a single crucial experiment can rarely be used to distinguish competing theories (Debrouwere & Rosseel, 2022), and perhaps ACs should be viewed as a way of adding nuance to debates rather than declaring a winner. Such a perspective might lower the intimidation factor surrounding ACs, but it also poses a challenge. Whereas science often incentivizes big, broad, “wow!” findings, ACs are likely to produce more subtle, nuanced findings, and so ACs might be punished in the peer review process. For this reason, collaborators might benefit most from conducting ACs in combination with registered reports (RRs), which are accepted for publication based on the methods prior to data collection. Scientific journals that aim to promote rigor above flashy but overstated findings might consider publishing RRs (and, in particular, RRs of ACs) exclusively for empirical papers.

Some ideas are undoubtedly inaccurate, and ACs are well-suited to expose such cases. However, none of our participants reported that their theory alone was proven incorrect. It is possible that scholars who suspect their theories may not withstand scrutiny are particularly hesitant to engage in ACs. In such cases, recruiting adversaries who are peripheral collaborators of proponents, rather than primary proponents, might allow relevant perspectives to converge, even if primary proponents opt out. Further, extensively documenting beliefs, methods, and their implications before conducting studies could promote accountability and make it more challenging for scholars to cling to their positions when their theory is proven incorrect. This discussion connects with our finding on the serendipitous nature of how ACs currently form and raises the question of whether a more systematic approach could improve their effectiveness. If journals or organizations standardized methods for identifying conflicts and assembling teams, they could expand the scale and impact of ACs, resolving more disputes, particularly those involving weaker theories. In an era of preprints, overburdened reviewers, and skepticism toward for-profit publishers, actively supporting such initiatives could help journals regain goodwill.

A surprising theme that emerged from many participants was an increase in skepticism toward behavioral science. As one scholar admitted, “I have become more convinced that a considerable number of experiments are designed in ways which are conducive to supporting an argument. It has made me more skeptical about the literature.” This heightened skepticism appears to stem from the process of ACs, which expose scholars to the unstated assumptions underlying research designs and highlight how differing perspectives can lead to contrasting inferences from the same results. Given this, ACs may be particularly valuable by incorporating prediction (Hofman et al., 2021) or focusing on solution-oriented problems (Watts, 2017). Projects with practical implications—such as informing policy or addressing societal challenges—offer a promising avenue for integrating adversarial perspectives while advancing both scientific rigor and societal impact, pushing behavioral science to be more worthy of trust.

While ACs hold clear promise, they also come with considerations, including limited scalability and potential effects on the pace of research. First, our participants noted that ACs were not suited to every scholar or topic; they work best when investigators possess the right temperament and when a genuine, well-defined disagreement exists. They should therefore be viewed not as method to revolutionize all of science, but as a targeted strategy for resolving select debates. Second, our participants noted that ACs demand greater upfront effort than typical collaborations and often produce more nuanced conclusions. This additional rigor may make researchers less willing to make strong claims and may slow the overall tempo of science. Whether this slower, more reflective progress is a drawback or a virtue will depend on one’s priorities for scientific advancement.

This study has several limitations. First, our sample is inherently biased, as it includes only scholars who have successfully completed an AC—a rare and challenging method. These participants may be predisposed to view ACs positively, potentially skewing our findings. Moreover, some failed ACs likely never reached public awareness, and so it is likely that we were unable to identify scholars who had the most negative experiences. Future research should make a larger call to identify these scholars to learn lessons on avoiding the worst outcomes. Second, our reliance on self-reported data limits the reliability of our conclusions. We did not directly measure shifts in beliefs, or the quality of the studies produced, leaving room for subjective interpretations and potential inaccuracies. Future research could address these limitations by surveying participants before and after their collaborations to capture belief changes more systematically and by incorporating more objective measures of study quality.

## Conclusion

The critical tradition in science carries a certain romanticism about bringing adversaries together to resolve debate. It reflects the ideal that scholars should be open to ideas from those they oppose, willing to hear them out, understand them fully, and, when disagreement remains, put it to the test. When we fail to engage with opposing perspectives, we risk isolating ourselves in intellectual silos, missing opportunities to contribute effectively to scientific knowledge. Although ACs have promise as a tool for advancing understanding, that promise remains largely underutilized, with few trials to learn from. By applying the lessons from this study, we hope to make ACs more accessible and impactful, encouraging scholars and journals to embrace their potential so we can better learn how to maximize their benefits and minimize the worst experiences and outcomes. We leave the final word to two of our participants, who provide advice for those considering this method: “Go for it,” and “Do it—and with an open mind!”.

## Data Availability

All data and code associated with this project are openly available at the following public repository: https://github.com/CalvinIsch/Reflections-on-AC.
